# Evaluating turnaround times for early infant diagnosis samples in Kenya from 2011-2014: A retrospective analysis of HITSystem program data

**DOI:** 10.1371/journal.pone.0181005

**Published:** 2017-08-10

**Authors:** Catherine Wexler, An-Lin Cheng, Brad Gautney, Sarah Finocchario-Kessler, Kathy Goggin, Samoel Khamadi, HITSystem Team

**Affiliations:** 1 University of Kansas Medical Center, Department of Family Medicine, Kansas City, Kansas, United States of America; 2 University of Missouri-Kansas City, School of Medicine, Kansas City, Missouri, United States of America; 3 Global Health Innovations, Kansas City, Missouri, United States of America; 4 Children’s Mercy Hospitals and Clinics, Health Services and Outcomes Research, Kansas City, Missouri, United States of America; 5 University of Missouri-Kansas City, School of Medicine, Kansas City, Missouri, United States of America; 6 University of Missouri-Kansas City, School of Pharmacy, Kansas City, Missouri, United States of America; 7 Kenya Medical Research Institute, Nairobi, Kenya; Yale University Yale School of Public Health, UNITED STATES

## Abstract

Long turnaround times (TAT) for the processing and posting of results of infant HIV DNA PCR samples can hinder the success of early infant diagnosis (EID) programs. The HITSystem is an eHealth intervention that alerts staff when services are overdue or results are delayed. We conducted a retrospective analysis of 3669 HIV-exposed infants enrolled in 15 Kenya hospital EID programs and three laboratories using the HITSystem from 2011–2014. We assessed mean and median TAT from when a sample was: 1) obtained to when it was shipped to the laboratory, 2) shipped to when it was received at the laboratory, 3) received to when a result was posted, and 4) the total time from obtaining the sample (step 1) to posting the result (step 3). TAT were compared by laboratory, clinic, year, and month of sample collection. 3625 infant samples had results posted by end of 2014. Mean TAT from sample collection to shipping was 5.2 days, from shipping to laboratory receipt was 2.0 days, and from laboratory receipt to result posting was 17.4 days. Altogether, it took an average of 24.7 days from sample collection until result posting. There was significant variation between laboratories, particularly in laboratory processing times (step 3). TAT showed a decreasing trend from 2011–2014, although TAT in December remained higher. Compared with other Kenyan studies, TAT in these HITSystem enrolled settings were shorter. Significant variation between laboratories, however, indicates the need to strengthen protocols and infrastructure to ensure that all laboratories can provide rapid, high-quality services.

## Introduction

Early identification and initiation of ART for HIV-infected infants can reduce mortality by up to 76% and slow disease progression [1] and are critical goals of early infant diagnosis (EID) programs. Prior to 2008, guidelines for infant HIV-testing in Kenya focused on the identification of HIV in infants who presented at pediatric or tuberculosis wards with symptoms [2]. However, in 2008, Kenya revised its guidelines to advocate for EID services to be provided to all HIV-exposed infants [3]. According to these revised guidelines, polymerase chain reaction (PCR) testing for HIV-exposed infants should occur when the infant is 6 weeks of age or at the first contact thereafter. Antibody testing should then occur at 9- and 18- months of age or 6 weeks after cessation of breastfeeding, with confirmatory PCR tests for infants with positive antibody tests [3].

In Kenya, the shift from using whole blood to using dried blood spots (DBS) in 2006 for infant PCR testing allowed for substantial scale-up of EID and made HIV testing possible even in remote areas [4]. For DBS, capillary blood samples are collected (usually by heel stick, minimizing the need for extensive phlebotomy training) on filter paper. DBS samples are stable at room temperature, require a minimal amount of blood from the infant, are easily transported, and pose minimal risk of infection [5,6]. The shift to using DBS, coupled with the new EID guidelines, contributed to a dramatic increase in the number of infants tested annually and resulted in a three-fold increase from 18,848 in 2008 to 59,413 in 2014 [7].

However, there are many points in the cascade of care where long turnaround times (TAT) delay the initiation of lifesaving ART for HIV-infected infants. Without point-of-care PCR testing technologies, samples must be shipped to one of seven centralized testing laboratories in Kenya [7]. This presents barriers for excessive TAT for infant samples to occur if the hospital delays routine shipment of samples to their designated central laboratory, if the courier service is delayed, or if samples are lost or damaged. Once the sample is received at the central laboratory, receipt is documented and the sample is processed [5]. Delays in sample processing at the laboratory can occur due to stock-outs of reagents, maintenance issues with the automated PCR testing equipment, or inadequate staffing to handle the volume of samples [8]. Once available, laboratories either ship paper-based results back to the hospital via commercial courier or use email and SMS printers and rely on clinical staff to record and communicate findings to mothers. This very manual process accounts for additional delays and each step presents a unique barrier for the sample or the result to be misplaced or mishandled, compromising the care, retention, and clinical outcomes for HIV-exposed infants [9].

Studies from Kenya suggest that delays at each step of this process vary substantially between settings and may create long turnaround times from when a sample is obtained to when the result is available to the clinician. It can take up to three weeks from the time a sample is obtained until it is received at the central laboratory and an additional one to three weeks from when it is received at the laboratory until the result is available to the hospital [5]. All said, it can take six to eight weeks from when a sample is collected to when the result is available at the hospital [5,10]. More recently, in standard of care settings, it took between 6.3 and 8.1 weeks from the time a sample was collected until the caregiver was informed of the infant’s result; however, the study did not directly report on the turnaround time between sample collection and hospital receipt of result [11]. Studies in other parts of East Africa report variable turnaround times, ranging from 9 days to 21 weeks [9,12,13].

The HITSystem is a web-based eHealth intervention that has three mechanisms that may improve EID sample turnaround time. First, the HITSystem tracks samples from the time that they are collected until the result is posted and generates electronic alerts when samples are delayed. These alerts allow providers to easily identify which samples do not have a result posted and follow up with the laboratory if s/he expects that the sample was lost, rejected, or forgotten. Second, if the sample was lost or rejected, a built in text messaging service allows providers to easily reach out to mothers and ask them to return to the hospital for a repeat blood draw. Third, once the sample is processed, results are entered directly into the HITSystem and become available to providers online. This eliminates any potential loss or delay that may occur when paper-based results are delivered via courier to the hospital, which is the standard of care. In a preliminary analysis conducted from one hospital and one laboratory from 2011–2012, TAT from DBS collection to the availability of results decreased from 4.08 weeks before HITSystem introduction to 2.48 weeks after HITSystem introduction [14].

The purpose of this study was to more fully describe TAT for EID samples collected as part of the HITSystem intervention in Kenya from 2011–2014. We report changes in TAT over time and identify points in the EID cascade most vulnerable to delays.

## Methods

### Study setting

We conducted a retrospective analysis of 3669 HIV-exposed infants enrolled in the HITSystem at 15 hospitals over a 3-year period, served by three central laboratories in Kenya. All hospitals and laboratories included in the study started using the HITSystem for EID between April 2011 and June 2014 (staggered start dates). The laboratories served at least one hospital (ranging from one to nine hospitals) that implemented the HITSystem. In 2015, labs 1, 2, and 3 processed an average of 1496, 595, and 1803 EID samples per month from all 361, 166, and 422 facilities served, respectively [7]. While all the labs were government run, they received varying support from outside funding agencies. All of the hospitals were government run and had Ministry of Health clinical staff implementing the HITSystem as an added service to their EID standard of care. All clinics and labs received training prior to HITSystem implementation. Training consisted of one 6-hour day for key hospital providers and one 3-hour day for key laboratory providers.

### Procedures

All initial DBS samples collected during this period were included in the analysis. Data are reported for the infant’s first PCR test result only. Since HIV DNA PCR tests at 9- and 18- months are only run for the small proportion of infants who have a positive antibody test, any confirmatory PCR run at these time points was not included in the analysis. Samples from HIV-exposed infants receiving routine care were collected, packaged, and shipped per the National EID guidelines and protocols established at each hospital. All clinics used a single courier system for sample shipment to lab. At sample collection, infants were enrolled in the HITSystem. A detailed description of the HITSystem has been published previously [11,15] but briefly, at enrollment infant and mother demographic data and the date of the initial DBS collection is entered into the HITSystem. The following dates are also recorded in the HITSystem: 1) date the sample was shipped from the hospital to the central laboratory, 2) date the sample was received at the laboratory, and 3) date the result was entered into the HITSystem. Automated alerts are sent to the laboratory technicians and the clinicians if any of these steps are not completed within a specified amount of time. This facilitates frequent communication and follow up between clinical staff and laboratory technicians.

### Ethical considerations

All mothers were informed about the HITSystem. Mothers provided oral informed consent to participate or were given the option to use only the paper-based registry without text messages or electronic alerts. Less than 1% declined to be entered in the HITSystem. Oral consent was deemed appropriate since participation involved minimal risk and a signed consent form would have been the only document outside of medical records that linked participants to their HIV status. The study was approved by the Institutional Review Board at the Kenya Medical Research Institute (SSC 1890).

### Measures

**Fig 1:**
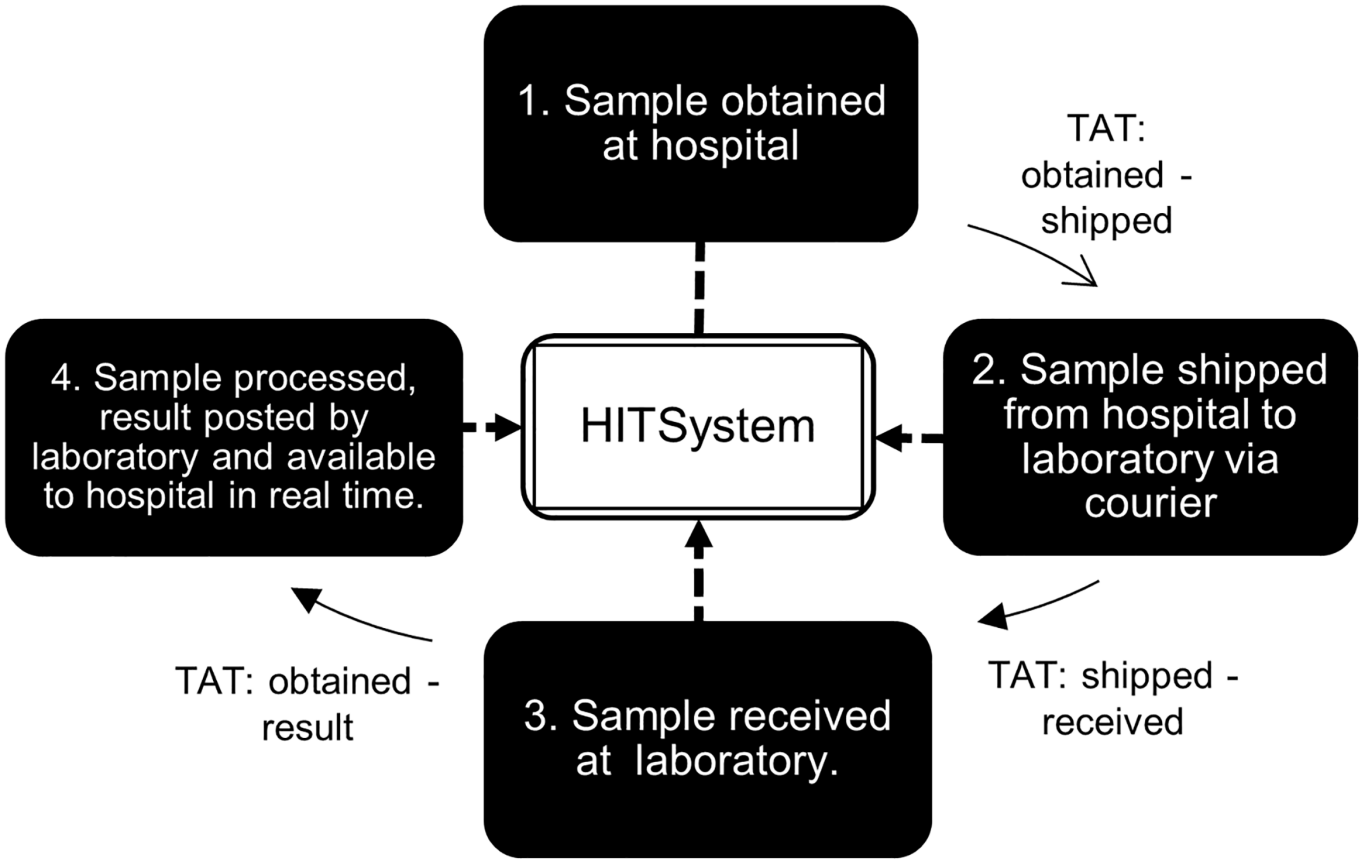
Flow of DBS sample processing from collection to results.

The primary outcome was overall TAT. This was defined as the number of days from when a sample was obtained to when the result was available to the hospital via the HITSystem. Overall TAT time was also broken down into three steps: 1) the number of days from when a sample was obtained to when a sample was shipped to the laboratory, 2) the number of days from when a sample was shipped until it was recorded as received at the laboratory, and 3) the number of days from when it was received at the laboratory until the result was posted in the HITSystem and became available for the clinician to see (Fig 1).

### Data analysis

We calculated mean and median days for each TAT by laboratory, hospital, year and month. Due to the natural distribution of TAT data (counted days), Poisson regression models were conducted to examine differences in overall TAT by laboratory and clinic.

## Results

Of the 3669 infant samples collected, 3625 (98.8%) had results posted by end of 2014. A total of 3407 (94%) samples were negative, 192 (5.3%) were positive, and 26 (0.7%) were indeterminate.

For all laboratories, the mean TAT from when a sample was obtained to when it was shipped was 5.2 days (median = 3; range = 0–698 days), the mean TAT from when a sample was shipped to when it was received at the laboratory was 2.0 days (median = 0; range = 0–253), and the mean TAT from when it was received at the laboratory to when a result was posted was 17.4 days (median = 13; range = 0–744). All said, it took a mean of 24.7 (median = 19, range = 0–776) days from when a sample was obtained until the result was available to the clinician. 76% of samples had results posted within 30 days of sample collection, 19% had results posted between 31 and 60 days of sample collection, and 5% had results posted over 60 days after sample collection.

The results from the Poisson regression models showed significant variation in the turnaround times between labs (Table 1). The mean TAT from obtained to shipped ranged from 1.7 days to 6.5 days, the mean TAT from shipped to received ranged from 0.3 days to 3.1 days, the mean TAT from received to results ranged from 12.5 days to 40.2 days, and the mean overall TAT ranged from 20.1 days to 41.9 days. It took Lab 3 nearly 2.5 times longer than the mean to process samples.

**Table 1:** Mean and median turnaround times in days by laboratory.

	N*	Mean Days (SD)	Median Days (Range)	P-value**
TAT: Obtained—Shipped to Lab				
All Labs	3582	5.2	3 (0–698)	<.0001
Lab 1	1823	6.5 (10.0)	5 (0–277)	
Lab 2	1388	4.6 (19.9)	2 (0–698)	
Lab 3	371	1.7 (3.8)	1 (0–63)	
TAT: Shipped—Received at Lab				
All Labs	3565	2.00	0 (0–253)	<.0001
Lab 1	1814	1.5 (9.3)	0 (0–238)	
Lab 2	1382	3.1 (15.9)	1 (0–253)	
Lab 3	369	0.3 (2.1)	0 (0–34)	
TAT: Received at Lab- Results Available				
All Labs	3539	17.4	12 (0–744)	<.0001
Lab 1	1797	17.6 (15.4)	14 (0–185)	
Lab 2	1442	12.5 (21.5)	10 (0–744)	
Lab 3	300	40.2 (26.6)	35 (0–126)	
TAT: Obtained—Results Available				
All Labs	3544	24.7	19 (0–776)	<.0001
Lab 1	1800	25.6 (21.6)	21 (0–438)	
Lab 2	1444	20.1 (33.2)	15 (0–776)	
Lab 3	300	41.9 (26.6)	36 (1–127)	

SD = standard deviation;

*N may differ between turnaround times due to missing data.

**P-Value calculated with Poisson Regression Models.

TAT by clinic also showed variation based on Poisson regression models (Table 2). The mean number of days from when a sample was obtained to when it was shipped to the laboratory ranged from 1.2 days to 11.7 days, with five of the fifteen hospitals averaging longer than one week to ship samples. Mean TAT from when a sample was shipped to the lab until it was received at the lab ranged from 0 days (received the same day it was shipped) to 4.2 days. Mean TAT from when a clinic obtained a sample to when the result was posted ranged from a low of 16.8 days to a high of 49.9 days.

**Table 2:** Mean and median turnaround times in days by clinic.

Clinic ID	TAT: Obtained to Shipped			TAT: Shipped to Received			TAT: Obtained to Results		
	N*	Mean (SD)	Median (Range)	N*	Mean (SD)	Median (Range)	N*	Mean (SD)	Median (Range)
1	371	1.2 (1.9)	1.0 (0–32)	371	3.5 (14.7)	1 (0–253)	371	16.8 (17.8)	14.0 (2–254)
2	372	1.7 (3.7)	1.0 (0–63)	369	0.3 (2.1)	0 (0–34)	300	41.9 (26.6)	36.0 (1–127)
3	123	1.9 (1.5)	1.0 (0–10)	123	4.2 (6.4)	3 (0–33)	124	22.6 (68.9)	13.5 (3–776)
4	132	2.7 (5.1)	1.0 (0–48)	132	4.1 (4.2)	3 (0–34)	153	18.9 (12.6)	15.0 (5–65)
5	49	4.7 (2.5)	5.0 (0–12)	49	3.1 (21)	0 (0–147)	49	20.1 (25.5)	14.0 (5–153)
6	639	5.1 (6.2)	4.0 (0–75)	640	0.3 (2.6)	0 (0–39)	630	22.8 (16.2)	19.0 (0–141)
7	112	5.5 (4.2)	5.0 (0–26)	112	2.6 (22.6)	0 (0–238)	113	20.3 (26.3)	15.0 (2–266)
8	55	5.9 (4.9)	6.0 (1–34)	55	0.02 (0.1)	0 (0–1)	55	16.8 (10.8)	14.0 (3–57)
9	654	6.1 (8.8)	5.0 (0–142)	648	2.5 (20.0)	0 (0–253)	657	20.5 (23.3)	16.0 (0–296)
10	24	6.3 (4.0)	6.0 (1–13)	24	0 (0)	0 (0–0)	24	18.3 (8.2)	16.0 (8–34)
11	834	7.3 (11.9)	5.0 (0–277)	831	2.5 (9.3)	0 (0–126)	827	28.3 (21.3)	25.0 (4–438)
12	17	7.9 (6.2)	6.0 (2–23)	17	1.8 (7.5)	0 (0–31)	16	49.9 (41.2)	39.5 (7–119)
13	12	8.8 (4.9)	7.5 (4–19)	12	0 (0)	0 (0–0)	12	18.5 (9.1)	14.5 (8–40)
14	78	11.7 (20.4)	6.0 (1–129)	73	0.8 (3.5)	0 (0–21)	73	37.8 (39.9)	30.0 (7–225)
15	110	11.9 (66.3)	3.0 (0–698)	109	3.1 (6.5)	1 (0–35)	140	25.3 (59.7)	18.0 (1–707)

SD = standard deviation;

*N may differ between turnaround times due to missing data.

**Fig 2:**
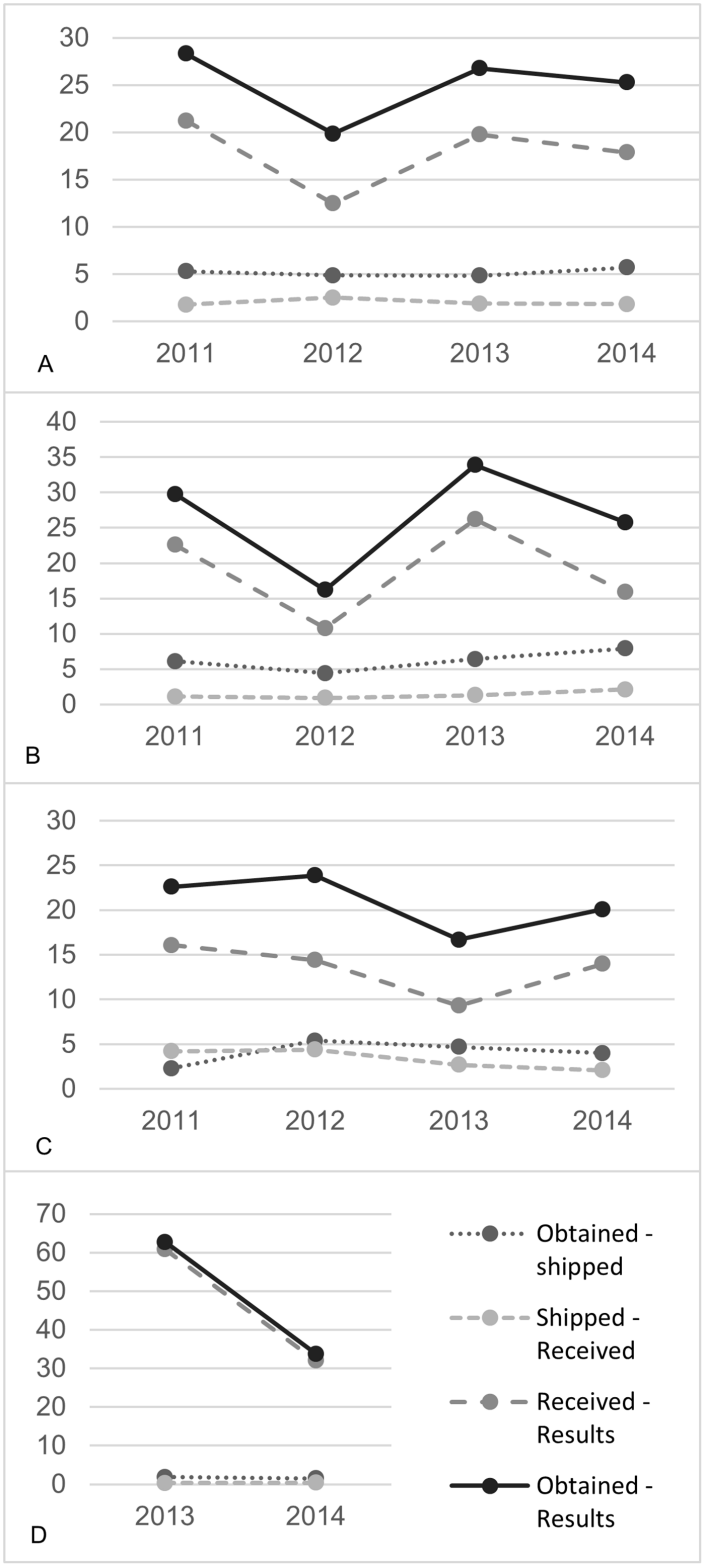
Mean turnaround times from 2011–2014. (A) Mean turnaround times by year for all labs. (B) Mean turnaround times by year for lab 1. (C) Mean turnaround times by year for lab 2. (D) Mean turnaround times by year for lab 3.

Mean TAT by year are reported in Fig 2. Labs 1 and 2 implemented the HITSystem in 2011, while Lab 3 implemented it in 2013. Across all labs, there was an overall decreasing trend from 2011 to 2014 (28.3 days from sample collection to result in 2011 to 25.3 days in 2014) (Fig 2A). This improvement was primarily driven by reductions in the time at the laboratory (i.e., from when sample received at laboratory until the test result was posted). While Labs 1 and 2 showed more modest improvements in mean TAT from when a sample was received to when a result was posted from 2011 to 2014 (from 29.8 days to 25.8 days and from 22.6 days to 20.1 days, respectively) (Fig 2B and 2C), Lab 3 nearly halved its mean TAT from when a sample was received to when results were posted (from 62.7 days to 33.8 days) between from 2013 to 2014 (Fig 2D).

While overall TAT from samples collected in January through November were similar (ranging from a low of 18.8 in June to a high of 26.0 in November), samples collected in December had a substantially longer overall TAT (42.1 days). This difference was driven primarily by longer TAT for processing and posting results at the laboratory.

## Discussion

This study provides the most comprehensive description of TAT for EID in Kenya, to date. The mean turnaround time from when a DBS sample was collected from an infant to when a result was posted was 24.7 days. This is faster than the 1.5–2 months reported in other studies in Kenya [10,11]. The TAT in this study was slightly longer than the 2.5 weeks observed in a preliminary analysis of the HITSystem at one hospital and one laboratory (Lab 2) by Gautney et al [14]. This analysis, however; includes 3 laboratories and 15 hospitals, increasing variability of TAT across hospitals and laboratories. While this increases the representativeness of these data it also contributed to the higher TAT reported here, partially due to the lack of consistent practices and capacity among sites. Clinic and laboratory characteristics, including varying levels of HITSystem ownership and varying rates of HITSystem adoption among facilities [15] and staffing levels and commitment at the different facilities may have contributed to differences in TAT. Furthermore, distance and road conditions between the hospital and the laboratory and the role of outside funding agency and varying EID volume among labs may have contributed to variations in sample shipment and sample processing times, respectively.

Time between receipt of sample at the laboratory to having a result posted accounted for the majority of total processing, followed by the time between a sample being obtained and being shipped. Shipping time was the shortest segment of the total TAT. In our study, the 17.4 day TAT from when a sample was received at the laboratory until a result was posted, reflecting efficiency of processing at the central laboratory, was slightly longer than the 13.5 days reported in Khamadi, et al [5], the only study we found from Kenya that reported on TAT between receipt of sample at the lab and availability of results to the hospital. However, data from Khamadi was collected from 2006–2008, prior to the substantial scale up of EID services in Kenya [5]. The increased TAT observed in this study could indicate an inadequate investment in laboratory infrastructure and personnel over the last several years to meet the growing demand for EID laboratory services and introduction of viral load testing for all HIV+ patients in 2014 [16]. Such improvements in the monitoring of HIV care will necessitate substantial scale up of laboratory capacity including purchases of additional laboratory diagnostic equipment, hiring and in-depth training of more laboratory technicians, and/or introduction of more health facilities with the capacity to run on-site testing. Without these investments, laboratories will be hard pressed to meet the growing demands for both EID and viral load sample processing. Point-of-care tests for EID is an emerging technology that has the potential to improve timely diagnosis by enabling same day results, significantly reducing the burden on laboratories, and improving pediatric HIV programming [17].

Delays at both the clinic and the laboratory contributed to the wide range of turnaround times observed in this study. Five of the 15 hospitals exceeded the 1 week target for shipping samples and excessive delays (> 60 days) to post test results were documented for 5% of the samples; indicating that improved oversight and management of such outliers could significantly improve overall TAT. We also observed some variability between sample shipment and receipt at lab between hospitals. While a single courier service is used across the country, distance from the hospitals to the designated central laboratory or road conditions could account for differences in TAT from shipping from hospital to receipt at laboratory. It may be important that courier companies with different capacities and efficiencies be identified to help improve efficiency of the sample transportation to testing laboratory.

Samples collected in December had the longest TAT, likely reflecting holiday travel and reduced availability of laboratory technicians. Staggering staff and support during the holiday season could help avoid lengthy delays. Since it is the excessive outliers that drive patient and provider dissatisfaction and can lead to repeat testing [18], improving TAT could minimize the need for repeat testing, and increase the percent of caregivers who collect their infant’s results in a timely manner [19]. Long TAT also delays infant ART initiation and can seriously compromise the health of HIV-infected infants. Tightening the range of TATs for EID samples is essential in order to optimize infant health outcomes, cost-effectiveness, and patient satisfaction.

In our sample, only 1.2% of DBS samples did not have a result posted by the end of the data collection period. This represents a significant improvement on results returned compared to the estimated 10%-63% of all infant samples in East Africa that never make it back to the hospital [9]. The HITSystem’s mechanisms for sample tracking and hospital/laboratory communication when test results are delayed may have contributed to the small number of unreturned results.

A number of limitations to the current study should be noted. First, as a retrospective review of HITSystem data, we did not have a comparison group. Thus, we were unable to comment on TATs in standard of care settings during the same period of time and whether these data represent an improvement to standard of care. A more rigorous randomized control trial to evaluate the HITSystem is underway [20], which will enable us to make such comparisons. Secondly, for this study, we are unable to know the exact reason for delays for individual samples or labs. However, we hope that through the ongoing randomized controlled trial to evaluate the HITSystem, [20] we will have more documentation on sample processing that will allow us to identify reasons for delays among specific samples, labs, and clinics. Thirdly, the interpretation of TAT changes over time is limited given the staggered start date of hospitals and their associated central laboratory over the study period. Two of the three laboratories began using the HITSystem in 2011, while Lab 3 began in 2013, and each showed improvement in the year after HITSystem implementation. Lastly, of the seven central laboratories in Kenya, the current study includes data for only three laboratories and therefore we are unable to comment on the TAT of the other four laboratories. Despite these limitations, these data provide an evaluation of TAT with a large sample size, inclusion of multiple central laboratories and hospitals, and the ability to observe changes over time.

## Conclusion

This study provides the most comprehensive and up to date description of turnaround time for EID in Kenya. While overall TAT showed modest improvement from 2011 to 2014, investments in infrastructure and laboratory personnel are needed to accelerate TAT, reduce the number of samples with excessive delays, and ensure that all laboratories providing EID diagnostic services are capable of keeping up with the increasing workload. While lack of a control group limits our ability to compare these results from HITSystem facilities to standard of care, this study provide promising evidence that the HITSystem can effectively monitor and manage TAT for EID samples by identifying TAT segments and time periods that need targeted support (i.e. sample processing at laboratories, staggered support during holiday months).
